# Diagnostic performance of the Abbott *RealTime* MTB assay for tuberculosis diagnosis in people living with HIV

**DOI:** 10.1038/s41598-021-96922-3

**Published:** 2021-09-29

**Authors:** Belén Saavedra, Edson Mambuque, Neide Gomes, Dinis Nguenha, Rita Mabunda, Luis Faife, Ruben Langa, Shilzia Munguambe, Filomena Manjate, Anelsio Cossa, Lesley Scott, Alberto L. García-Basteiro

**Affiliations:** 1 PhD Program in Medicine and Translational Research, Universitat de Barcelona, Barcelona, Spain; 2 Centro de Investigação Em Saude de Manhiça (CISM), Maputo, Mozambique; 3 ISGlobal, Hospital Clínic - Universitat de Barcelona, Barcelona, Spain; 4 Manhiça Health Research Hospital, Ministry of Health, National Tuberculosis Control Program, Maputo, Mozambique; 5 Department of Molecular Medicine and Haematology, School of Pathology, and iLEAD, Faculty of Health Sciences, Wits Health Consortium, University of the Witwatersrand, Johannesburg, South Africa

**Keywords:** Infectious-disease diagnostics, Tuberculosis

## Abstract

Strengthening tuberculosis diagnosis is an international priority and the advocacy for multi-disease testing devices raises the possibility of improving laboratory efficiency. However, the advantages of centralized platforms might not translate into real improvements under operational conditions. This study aimed to evaluate the field use of the Abbott RealTime MTB (RT-MTB) and Xpert MTB/RIF assays, in a large cohort of HIV-positive and TB presumptive cases in Southern Mozambique. Over a 6-month period, 255 HIV-positive TB presumptive cases were consecutively recruited in the high TB/HIV burden district of Manhiça. The diagnostic performance of both assays was evaluated against two different reference standards: a microbiological gold standard (MGS) and a composite reference standard (CRS). Results from the primary analysis (MGS) showed improved sensitivity (Se) and reduced specificity (Sp) for the Abbott RT-MTB assay compared to the Xpert MTB/RIF (RT-MTB Se: 0.92 (95% CI: 0.75;0.99) *vs* Xpert Se: 0.73 (95% CI: 0.52;0.88) *p* value = 0.06; RT-MTB Sp: 0.80 (0.72;0.86) *vs* Xpert Sp: 0.96 (0.92;0.99) *p* value < 0.001). The lower specificity may be due to cross-reactivity with non-tuberculous mycobacteria (NTMs), the detection of non-viable MTBC, or the identification of true TB cases missed by the gold standard.

## Introduction

Tuberculosis (TB) remains one of the leading causes of death and disease worldwide. The World Health Organization (WHO)'s End TB Strategy has set ambitious goals to be achieved by 2035: 90% reduction in TB incidence and 95% reduction in mortality compared with levels in 2015^1^. In 2019, approximately 10 million people were estimated to be infected with TB and became ill^1,2^. Efforts to accelerate TB elimination rely on several factors, but efficient and timely diagnosis of the disease is of utmost importance^3^. In recent years, considerable progress has been made in improving global case detection rates, although the gap between the reported and estimated number of cases is still unacceptable. Around 3 million incident cases were not diagnosed in 2019 due to under-reporting and/or under-diagnosis^1^. Therefore, increasing case detection and strengthening the diagnostic cascade remains a priority. For that purpose, National Control Programmes need to foster laboratory networks by equipping them with rapid and accurate diagnostic technologies^4,5^.

In 2018, the WHO endorsed a list of essential novel tests for diagnosing TB^6^. Nucleic acid amplification tests (NAAT) have substantially advanced the investigation of *Mycobacterium tuberculosis complex* (MTBC) and have been recognized as promising tools in closing the gap between diagnosis and treatment. Not only are NAAT more sensitive and faster than traditional assays, they also allow more accurate and rapid resistance detection^7–9^. However, their application is limited due to lack of trained staff and availability of proper infrastructure in low resource settings^8^. In the last decade, the endorsement of the Xpert MTB/RIF (hereinafter Xpert) as an initial test for TB investigation has radically changed the landscape of TB diagnosis^5^. This cartridge-based molecular assay was designed for the Cepheid's GeneXpert System (module-based), which allows the rapid detection of the MTBC DNA and also detects more than 95% of mutations associated with rifampicin resistance^10,11^. Besides, this flexible and user-friendly platform, offers a complete range of tests for the diagnostic of many other infectious diseases^12^.

However, the molecular TB diagnostic pipeline is broader^8,13^. Recently, through the release of rapid communication, WHO made new recommendations on the use of molecular assays as initial tests for TB diagnosis (Xpert MTB/RIF, Xpert Ultra, and TrueNat MTB have shown to have good performance for the detection of TB and rifampicin resistance detection^9^). Additionally, in a Technical Expert Consultation Report, the WHO also evaluated the use of four centralized assays for similar purposes^13^.

The automated RealTime MTB assay has been developed by Abbott Molecular (Chicago, IL, USA; hereinafter RT-MTB) for the qualitative detection of MTB complex and genetic variants of rifampicin (RIF) and isoniazid (INH) resistance using the RT-MTB RIF/INH reflex option. The assay has been designed for testing on the Abbott *m*2000 System (*m*2000*sp* and *m*2000*rt*)^14,15^. These are high-throughput instruments, widely used for HIV-1 viral load monitoring, and a broad range of tests for other pathogens, including the recently discovered SARS-CoV-2^16^. A major challenge for diagnostic laboratories is to enhance their operational productivity by integrating and simplifying laboratory techniques. The advocacy for high-capacity, multi-disease testing devices, mainly in settings with limited resources, provides the option of improving laboratory efficiency and cost-saving.

Early studies on the performance evaluation of the RT-MTB assay have shown varying results in both, clinical and in vitro analysis (Supplementary material, S1)^14,17–24^. While acceptable sensitivity and specificity values were achieved, few of these studies were conducted in high TB burden countries, and none of them were carried out on a large cohort of HIV-1 positive patients. People living with HIV (PLHIV) are one of the most vulnerable populations for TB disease, often paucibacillary and in whom other tests, such as Xpert, have shown lower sensitivity^5^. It is, therefore, crucial to use a diagnostic platform that will adequately identify MTB in individuals co-infected with HIV.

The aim of this study is to assess the diagnostic performance of the RT-MTB assay in a unique cohort of PLHIV in a high TB and HIV burden country^25,26^. To our knowledge, this is the first study that compares the performance analysis of RT-MTB and Xpert MTB/RIF in such a cohort using 2 different reference standards.

## Materials and methods

### Study design and setting

This is a prospective evaluation of the diagnostic performance of the Abbott RT-MTB and its RT-MTB RIF/INH assays. The study was conducted in the district of Manhiça, Maputo province, a rural area 80 km away from the capital with a population of approximately 190,000 inhabitants^27^. The HIV prevalence estimate in this district was 39.2% in 2012^28^ and the latest published incidence rate of lab-confirmed TB among PLHIV (aged 18–47 years) is 847 per 100,000 population^29^.

Consecutive HIV-positive adults identified at the Manhiça District Hospital (HDM) were screened for any symptom compatible with TB, as recommended by WHO guidelines ^30^ between July to December 2016. Patients with at least one of the following symptoms: cough for any duration, hemoptysis, night sweats, fever or unintentional weight loss, were offered to participate in the study. Those who self-reported having received TB treatment within the previous 6 months were excluded. All samples were tested at the *Centro de Investigação em Saúde de Manhiça (CISM)* laboratory.

### Study procedures

Participants were enrolled at the clinic of the National Tuberculosis Programme (NTP) in Manhiça village after informed consent and data were collected through specific questionnaires. Blood samples were obtained for viral load testing using the automated RealTi*m*e HIV-1 Assay (Abbott Molecular). If participants had no recent CD4 results (documented within the last 3 months), TruCount blood tubes (Becton Dickinson Biosciences, San Jose, CA) were also collected to determine T-cells counts by flow cytometry.

As part of the TB diagnostic workup, participants provided two sputum samples, which were received the day following enrolment at the CISM laboratory. Sputum induction was performed for individuals that were unable to provide sputum spontaneously. Clinically diagnosed or laboratory-confirmed TB patients were managed according to the National Tuberculosis Programme (NTP) guidelines and were started on TB treatment. In case of discordant results between RT-MTB (positive) and the standard of care (culture and Xpert negative), patients were contacted for re-evaluation and were requested to provide a third sputum sample to aid the decision of treatment iniciation. All participants were scheduled for a follow-up visit 2 months after enrolment. Those who did not attend the clinic on day 60 were contacted by telephone and interviewed.

### Laboratory procedures

All diagnostic tests were performed in a TB Biosafety level 3 (BSL-3) laboratory, which is subject to external quality control and ISO certification.

**Figure 1:**
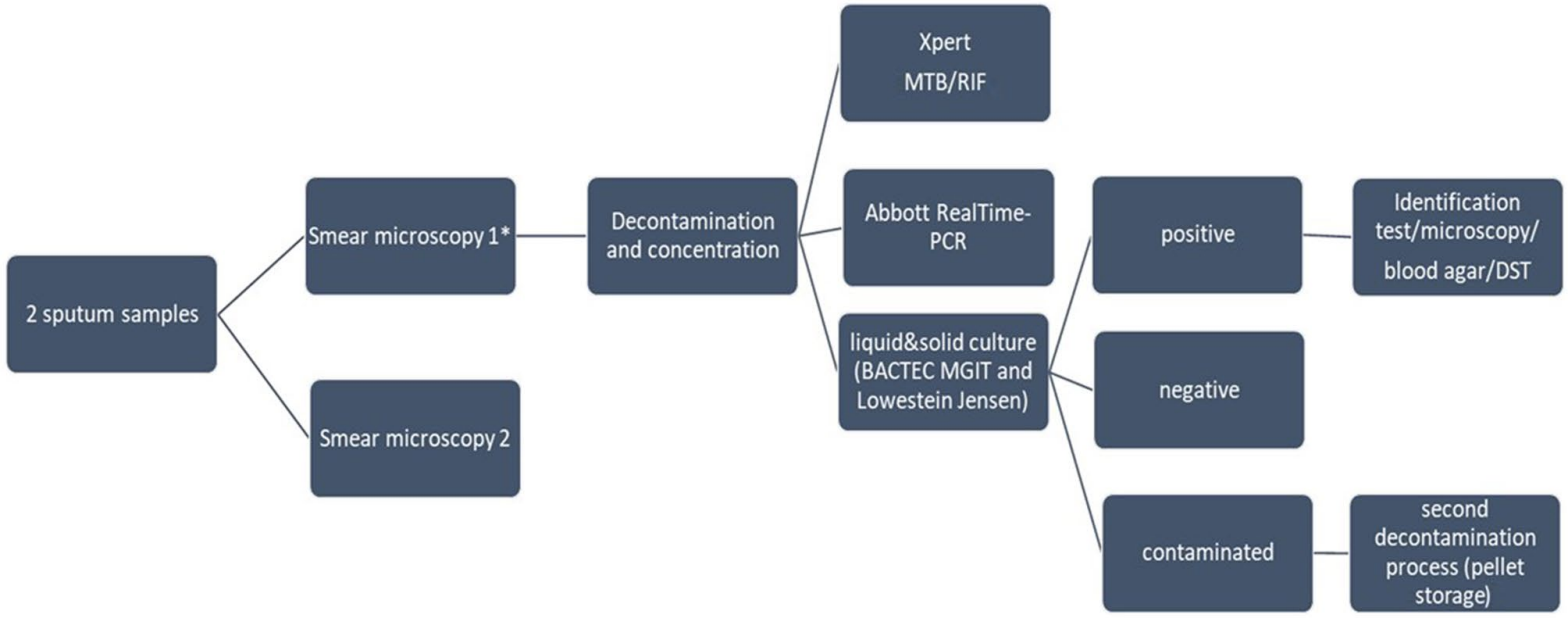
Laboratory flowchart. (*) The best quality sample was used to perform molecular tests.

Smear microscopy for both sputum specimens was performed by Ziehl Neelsen (ZN) staining. Results were reported as negative or on a scale of positive grades according to international standards. For each participant, specimens with the best quality were decontaminated using the Kubica method ^31^ and the resuspended pellet was used for all tests to compare diagnostic accuracy among homogeneous specimens (Fig. 1). For liquid cultures, 500 µl of the decontaminated pellet were inoculated into Mycobacterium Growth Indicator Tubes (MGIT) liquid medium and incubated in the Bactec MGIT 960 mycobacterial detection instrument (Becton Dickinson Microbiology System, USA) according to manufacturer´s instructions. Additionally, 200 µl were cultured in BD Lowenstein Jensen solid medium. After 42 days (for liquid culture) or 8 weeks (for solid culture) without growth, samples were classified as negative. In the case of positive results, MTBC was confirmed using ZN staining and BD TB Identification test (Becton Dickinson Microbiology System, USA). Phenotypic drug susceptibility tests (DST) were conducted on all positive cultures.

Molecular assays were also performed according to the manufacturer´s instructions. Briefly, 500 µl of the sediment were processed by Xpert MTB/RIF after mixing with the specific Sample Reagent in a ratio of 1:3. Two milliliters of the mixture were transferred into the cartridge. Results were obtained in a maximum of 2 h. Similarly, for the RT-MTB assay*,* 500 µl of the decontaminated specimen were mixed with the specific inactivation reagent (LabMate, South Africa UK) in a ratio of 1:3 and were incubated for 1–48 h. Automated DNA extraction and sample preparation (addition of master mix) were performed on the *m*2000*sp* instrument followed by real-time PCR and automated result interpretation using the *m2000rt* instrument. Residual extracted nucleic acid aliquots from *m*2000*sp* deep well plates were stored at − 25 to − 15 °C for up to 90 days for drug susceptibility testing. Samples identified as MTBC positive by the RT-MTB were automatically selected by the “reflex” software on the *m*2000*sp* and tested for RIF and INH resistance using the RT-MTB RIF/INH assay^32^ Residual pellets were stored at − 25 to − 15 °C until the end of the study.

### Statistical analysis

The statistical analysis was performed using R version 3.5.2 (The R Foundation for Statistical Computing Platform). Figures and tables were created using Excel Microsoft Office.

Baseline characteristics of individuals were reported using mean and standard deviation, proportions, or median and interquartile range, depending on the variable type. The diagnostic performance of the RT-MTB and Xpert MTB/RIF was assessed through calculation of sensitivity (Se) and specificity (Sp), negative and positive predictive values (NPV and PPV) of the tests (with binomial distribution 95% confidence intervals).

Two different cohorts were evaluated: the per-protocol cohort (those who completed the follow-up visit at month 2) and the intention-to-treat cohort (all patients initially enrolled irrespective of having follow-up visit). For the per-protocol cohort, we conducted a primary analysis using aggregated results on solid and liquid MTBC culture as a gold standard (hereinafter microbiological gold standard, MGS). Patients were classified as “microbiologically confirmed” if either liquid or solid culture was positive. If both cultures were contaminated, they were excluded from the analysis. Non-tuberculous mycobacteria (NTM) growths were considered negative for MTBC. Patients in whom culture or NAAT assays results were not available (contamination or invalid results) were excluded from the analysis. For a secondary diagnostic test evaluation, a composite reference standard (CRS) was made by combining culture results and clinical information on treatment initiation (blinded to RT-MTB results). Lastly, in our “intention to treat” cohort we compared results to the microbiological reference standard.

The McNemar’s test with continuity correction or the Exact nominal Symmetry Test when discordant cells had low counts, were used to evaluate a systematic difference between the performance of the Xpert and RT-MTB and against both reference standards.

### Ethical considerations

This study was submitted to all relevant ethics boards. The protocol was approved by the CISM’s Internal Scientific Committee, the CISM’s Internal Bioethics Committee (CIBS – Comité Institucional de Bioética para a Saúde) and the National Bioethics Committee (CNBS—Comité Nacional de Bioética para a Saúde) with Ref Number 101/CNBS/2016. All individuals provided written informed consent to participate. The study methods were carried out under the relevant guidelines and regulations established by the National Bioethics Committee at the Ministry of Health.

## Results

During the 6-month study period, 255 HIV-positive and TB presumptive individuals were enrolled following informed consent. Of these, only 227 patients provided sputum samples for TB investigation. Eleven participants were excluded due to the unavailability of test results and therefore, a total of 216 patients were included in the final analysis (Fig. 2).

**﻿Figure 2:**
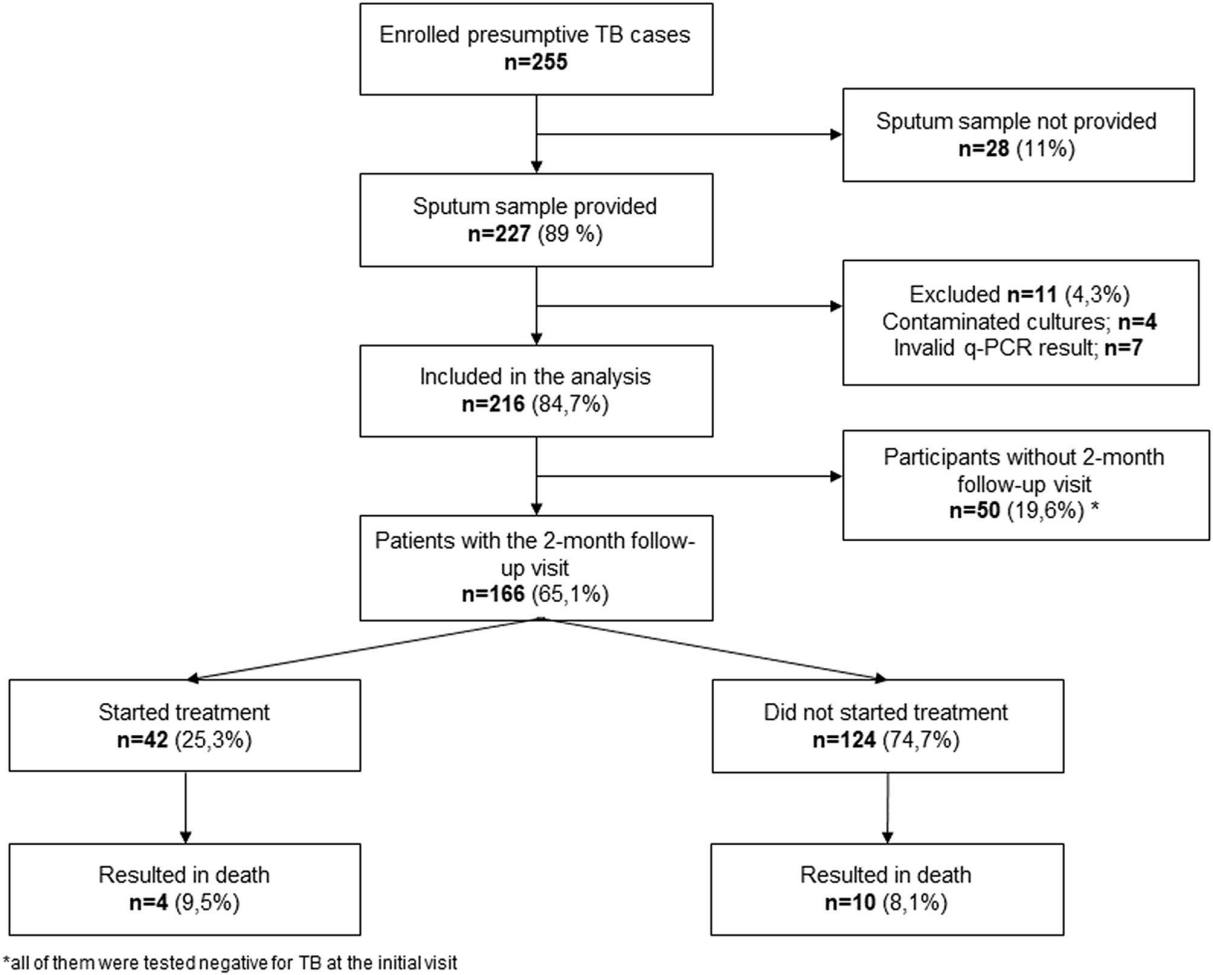
Participant enrolment flowchart. *Participants tested negative for TB at the initial visit.

The sociodemographic and clinical characteristics of all participants were assessed (Table 1). The median age was calculated as 27 years [IQR 23;35], with 51.4% of them (111/216) identified as female and 58.3% (126/216) were on antiretroviral therapy (ART).

**Table 1:** Per-protocol cohort. Characteristics of individuals included in the analysis (n = 216).

Type of variable	Variable	n (%)	
Sociodemographic	Sex		
	*Female*	111 (51.4)	
	*Male*	105 (48.6)	
	Age	median [IQR]^1^ 27.0 [23;35]	
HIV-related	CD4 data available	205 (94.9)	
	CD4 count (cells/mm3)	median [IQR]: 277 [84;498]	
	ART^2^ information available	215 (99.5)	
	Participants on ART	126 (58.3) ^*3*^	
		*Viral RNA not detected *^*4*^	66 (52.4)
		*Viral RNA detected*	57 (45.2)
		*Viral load (log(10)copies/ml)*	median [IQR]: 4.32 [3.54;5.27]
	Participants not on ART	89 (41.2)	
		*Viral RNA not detected*	9 (10.1)
		*Viral RNA detected*	74 (83.1)
		*Viral load (log(10)copies/ml)*	median [IQR]: 4.90 [4.26;5.53]
TB-related	TB symptoms		
	*Cough*	211 (97.7); median days [IQR]: 8 [7;30]	
	*Fever*	80 (37.0) median days [IQR]: 7 [5;15]	
	*Weight loss*	116 (53.7)	
	*Night sweat*	115 (53.2)	
	*Loss of appetite*	92 (42.6)	
	Previous TB	13 (6.0)	
	TB Treatment initiation	42 (19.4)	
	*Microbiologically confirmed (composite reference standard)*	32/42 (76.2)	
	Deaths	14 (6.4)	
	*On ART*	6/14 (42.9)	

Figures have been calculated based on the total number of participants with baseline visit (n = 216) ^1^*IQR* interquartile range; ^2^*ART* antiretroviral therapy. ^3^3 missing values for viral load results; ^4^The limit of detection for the Abbott Realtime HIV-1 assay was 150 copies/ml.

### Per-protocol cohort

From 216 patients, 23.1% (50/216) did not attend the clinic for the 2-month follow-up visit and were excluded from the per-protocol analysis. All of them tested negative for TB at the initial visit, and based on NTP records, they did not start treatment during the study period. Of the final cohort of 166 patients, 15.6% (26/166) were TB positive by aggregated culture results, 8.4% (14/166) were smear positive, 11.4% (19/166) were positive by Xpert and 14.4% (24/166) by RT-MTB (Table 2).

**Table 2:** Absolute test results for the per-protocol cohort (n = 166) for the intention-to-treat cohort (n = 216).

*MGS*^§^	Overall		Smear negative (n = 151)		Smear positive (n = 15)	
	MTBC* positive	MTBC negative	MTBC positive	MTBC negative	MTBC positive	MTBC negative
**Per-protocol cohort (n = 166)**						
Smear microscopy +	14/26	1/140	–	–	–	–
Xpert MTB/RIF +	19/26	5/140	5/12	4/139	14/14	1/1
RT-MTB +	24/26	28/140	10/12	27/139	14/14	1/1

*CRS*^*#*^	TB **case positive	TB case negative	TB case positive	TB case negative	TB case positive	TB case negative
Smear microscopy +	15/46	0/120	–	–	–	–
Xpert MTB/RIF +	24/46	0/120	9/31	0/120	15/15	0/0
RT-MTB +	30/46	22/120	15/31	22/120	15/15	0/0

*MGS*	Overall		Smear negative (n = 201)		Smear positive (n = 15)	
	MTBC positive	MTBC negative	MTBC positive	MTBC negative	MTBC positive	MTBC negative
**Intention to treat cohort (n = 216)**						
Smear microscopy +	14/27	1/189	–	–	–	–
XPERT MTB/RIF +	19/27	5/189	5/13	4/188	14/14	1/1
RT-MTB +	24/27	37/189	10/13	36/188	14/14	1/1

*MTBC Mycobacterium tuberculosis complex; **TB tuberculosis; ^§^*MGS* microbiological gold standard (results on aggregated culture), ^#^*CRS* composite reference standards (by combining microbiological results and clinical information on treatment initiation).

#### Primary analysis, (MGS)

Table 3 provides comprehensive results on the performance of the Xpert and RT-MTB assays for both study cohorts using aggregated culture as the gold standard. Overall, Xpert sensitivity was lower than RT-MTB, (0.73 (95% CI: 0.52–0.88) *vs* 0.92 (95% CI: 0.75–0.99) *p* value = 0.06) respectively. In both tests, sensitivity decreased among smear negative patients, maintaining the improved but weak statistical evidence on the performance of RT-MTB over Xpert (*p* value = 0.063). Conversely, there was strong evidence of differences in specificity and PPV between Xpert and RT-MTB in all cases (i.e. overall Xpert Sp: 0.96 (0.92;0.99) *vs* RT-MTB Sp: 0.80 (0.72;0.86), *p* value < 0.001; overall PPV: 0.79 (0.58;0.93) *vs* 0.46 (0.32; 0.61)).

**Table 3:** Primary analysis. Diagnostic test values using aggregated culture results as the reference standard. Comparison between the per-protocol and the intention-to-treat cohort.

	Per-protocol analysis; Microbiological gold-standard (n = 166)								Intention to treat analysis; Microbiological gold standard (n = 216)							
	Sensitivity (95%CI*)	Specificity (95%CI)			PPV^§^ (95%CI)	NPV^*#*^ (95%CI)			Sensitivity (95%CI)	Specificity (95%CI)			PPV (95%CI)	NPV (95%CI)		
**Overall**																
Smear microscopy	0.54 (0.33;0.73)	0.99 (0.96;1.00)			0.93 (0.68;1.00)	0.92 (0.87;0.96)			0.52 (0.32;0.71)	0.99 (0.97;1.00)			0.93 (0.68;1.00)	0.95 (0.86;0.97)		
Xpert MTB/ RIF	0.73 (0.52;0.88)	0.96 (0.92;0.99)			0.79 (0.58;0.93)	0.95 (0.90.0.98)			0.70 (0.50;0.86)	0.97 (0.94;0.99)			0.79 (0.58;0.93)	0.96 (0.92; 0.98)		
RT-MTB	0.92 (0.75;0.99)	0.80 (0.72;0.86)			0.46 (0.32; 0.61)	0.98 (0.94;1.00)			0.89 (0.71;0.98)	0.80 (0.74;0.86)			0.39 (0.27;0.53)	0.98 (0.94;1.00)		
	*p* value = 0.06	*p* alue < 0.001							*p* value = 0.06	*p* value < 0.001						
**Smear negative**																
Xpert MTB/ RIF	0.42 (0.15;0.72)	0.97 (0.93;0.99)			0.56 (0.21;0.86)	0.95 (0.90;0.98)			0.38 (0.14;0.68)	0.98 (0.95;0.99)			0.56 (0.21;0.86)	0.96 (0.92;0.98)		
RT-MTB	0.83 (0.51;0.98)	0.81 (0.73;0.87)			0.27 (0.14;0.44)	0.98(0.94;1.00)			0.77 (0.46;0.95)	0.81 (0.74;0.86)			0.22 (0.11; 0.36)	0.98 (0.94;1.00)		
	*p* value = 0.06	*p* value < 0.001							*p* value = 0.06	*p* value < 0.001						
**Smear positive**																
Xpert MTB/ RIF	1.00 (0.77;1.00)	–			0.93 (0.68;1.00)	–			1.00 (0.77; 1.00)	–			0.93 (0.68;1.00)	–		
RT-MTB	1.00 (0.77;1.00)	–			0.93 (0.68;1.00)	–			1.00 (0.77; 1.00)	–			0.93 (0.68;1.00)	–		

*CI confidence interval; ^§^*PPV* positive predictive value; ^*#*^*NPV* negative predictive value.

#### Secondary analysis, (CRS)

The diagnostic test values of the per-protocol cohort using the CRS are provided in Table 4. From Table 2, 27.7% (46/166) of participants were diagnosed with TB. Twenty-six of them tested positive for MTBC culture while the remaining twenty individuals started TB treatment per clinical criteria. The overall sensitivity of the three assays dropped when the results of the CRS and MGS were compared. As seen previously, RT-MTB and Xpert did not show systematic differences in sensitivity, except for smear negative patients (RT-MTB Se: 0.48 (0.30;0.67) *vs* Xpert Se: 0.29 (0.14;0.48) *p* value = 0.05).

**Table 4:** Per-protocol  secondary analysis. Diagnostic test values using the composite reference standard (Tuberculosis treatment and microbiological results) n = 166.

	Composite reference standard (TB treatment initiation n = 166)			
	Sensitivity (95%CI*)	Specificity % (95%CI)	PPV^§^ (95%CI)	NPV^*#*^ (95%CI)
**Overall**				
Smear microscopy	0.33 (0.20;0.48)	1.00 (0.97; 1.00)	1.00 (0.78; 1.00)	0.79 (0.72; 0.86)
Xpert MTB/RIF	0.52 (0.37;0.67)	1.00 (0.97; 1.00)	1.00 (0.86; 1.00)	0.85 (0.77; 0.90)
RT-MTB	0.65 (0.50; 0.79)	0.82 (0.74; 0.88)	0.58 (0.43; 0.71)	0.86 (0.78; 0.92)
	*p* value = 0.1	*p* value < 0.001		
**Smear negative (n = 151)**				
Xpert MTB/RIF	0.29 (0.14;0.48)	1.00 (0.97; 1.00)	1.00 (0.66;1.00)	0.85 (0.77;0.90)
RT-MTB	0.48 (0.30;0.67)	0.82 (0.74; 0.88)	0.41 (0.25;0.58)	0.86 (0.78;0.92)
	*p* value = 0.05	*p* value < 0.001		
**Smear positive (n = 15)**				
Xpert MTB/RIF	1.00 (0.78; 1.00)	–	1.00 (0.78; 1.00)	–
RT-MTB	1.00 (0.77;1.00)	–	0.93 (0.68;1.00)	–

*CI confidence interval; ^§^*PPV* positive predictive value; ^*#*^*NPV* negative predictive value.

The 22 RT-MTB false-positive subjects were further characterized. Based on culture results, 27.3% (6/22) were found to be positive for NTM. Three participants died after the 2-month study follow-up, four had been previously treated for TB and two of them had completed treatment only 1 year before enrolment. Follow-up results are provided in Supplementary material (S2). Additionally, cross-contamination into the Abbott *m2000sp* instrument  was assessed. No amplification of MTBC was detected in surrounding samples of those theoretically considered false-positive.

**Figure 3:**
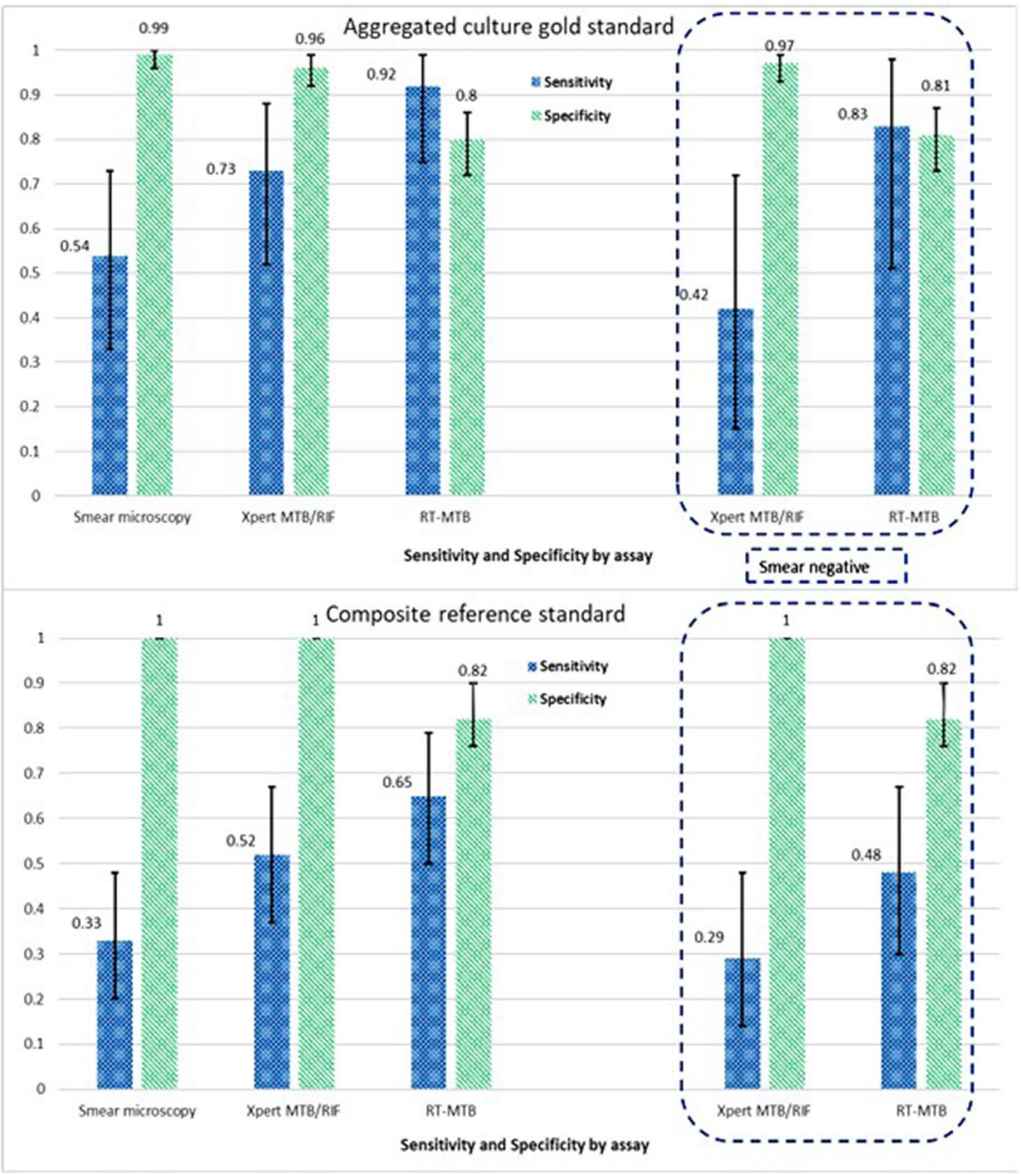
Bar chart illustrating sensitivity and specificity values by reference standard. On the left-side overall values are displayed; on the right-side, test values among smear negative patients. Line chart represents 95% confidence intervals.

**Figure 4:**
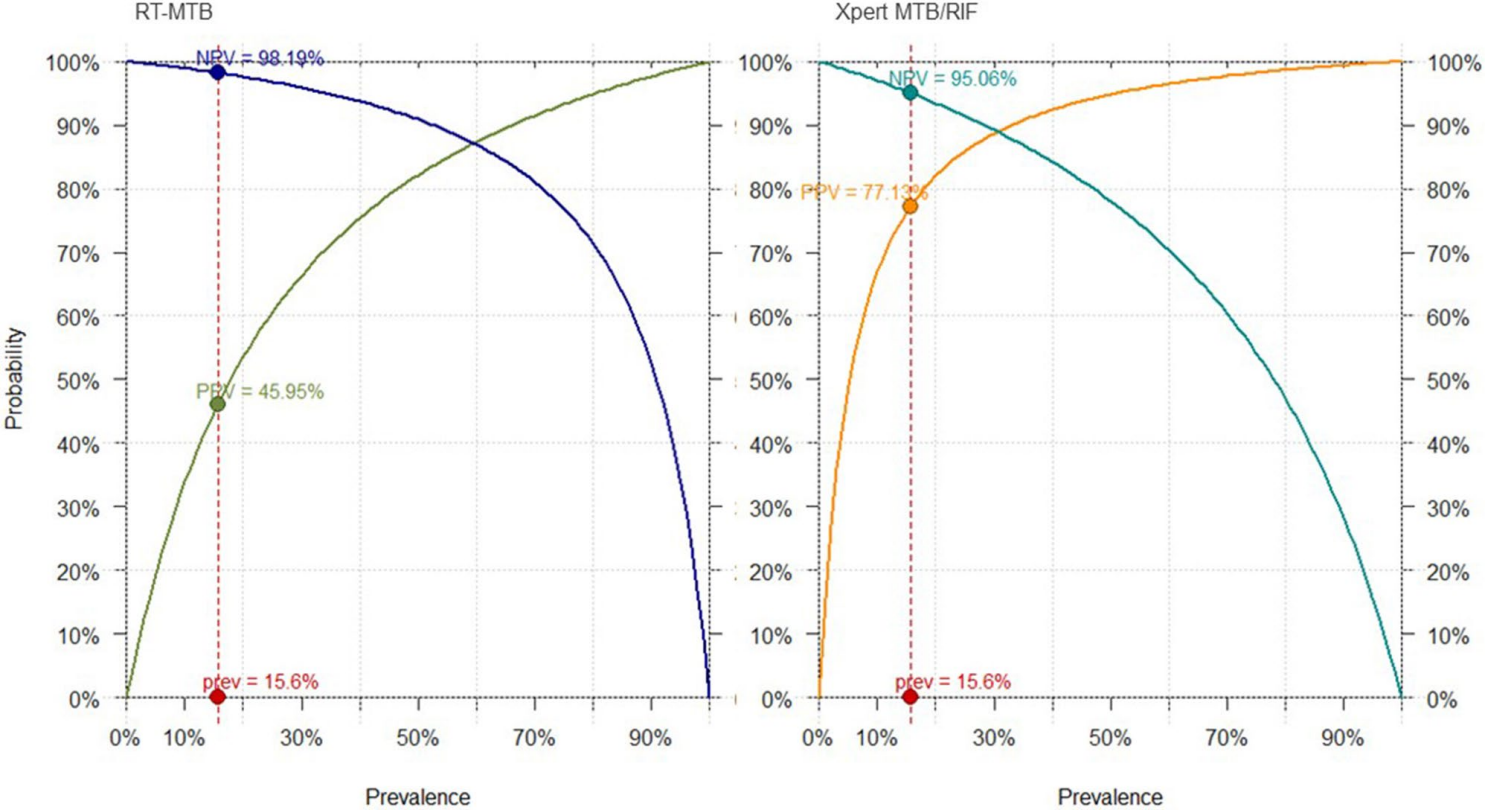
Xpert and RT-MTB PPVs and NPVs by pre-test probability. The prevalence applied has been calculated relying on the microbiological reference standard. *NPV* negative predicted value, *PPV *positive predicted value, *Pre* prevalence.

**Figure 5:**
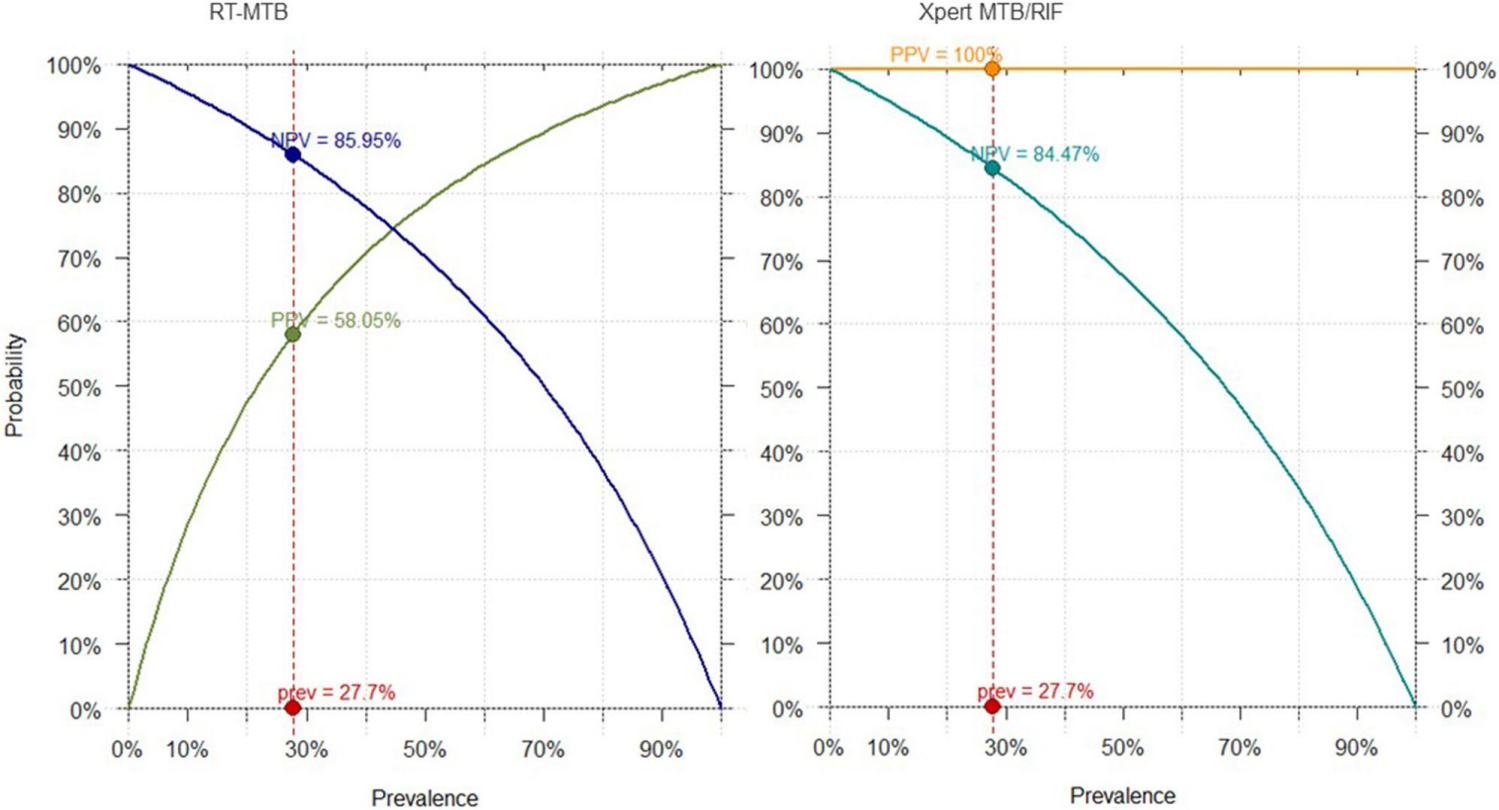
Xpert and RT-MTB PPVs and NPVs by pre-test probability. The prevalence applied has been calculated relying on the composite reference standard. *NPV* negative predicted value, *PPV* positive predicted value, *Pre* prevalence.

Figure 3 illustrates test values and 95%CI by test and reference standard. Figures 4 and 5 shows projections of positive predictive values (PPV) and negative predictive values (NPV) by pre-test probability.

### Intention-to-treat cohort

The overall test performance of the intention-to-treat cohort was found to be similar to the per-protocol cohort of patients, as shown in Table 3. Overall, we did not find systematic differences in sensitivity between assays although Xpert´s specificity was higher in all cases (overall Xpert Sp: 0.97 (0.94;0.99) *vs* RT-MTB: 0.80 (0.74;0.86) *p* value < 0.001). Findings were also stratified by smear microscopy and final data is detailed in Tables 2 and 3.

### Threshold cycle number values for RT-MTB and RT-MTB RIF/INH results

Overall, the mean threshold cycle number (Cn) was 28.42 (SD 7.25), and 35.67 (SD 18.45) for false-positive samples. Concerning drug susceptibility, Xpert yielded 100% valid results (24 over 24 Xpert-positives results), compared to 34.6% (18 out of 52 positives by RT-MTB). Neither Xpert nor RT-MTB detected gene targets for rifampicin resistance, whereas markers for isoniazid monoresistance were identified in 2 patients by RT-MTB RIF/INH assay. From those samples without RT-MTB RIF/INH results (35/52), 21 of them proved below the limit of detection (LoD). When comparing with phenotypic DST, 57.7% (15/26) of culture positive cases got a resistance profile from RT-MTB RIF/INH assay. Fourteen of these results (14/15, 93.4%), agreed with the phenotypic DST. The only one discordant sample harbored a mutation related to low level of isoniazid resistance which did not translate into phenotypic resistance.

### Operational characteristics and challenges

Our laboratory follows rigorous quality control procedures and instrument track records were registered and detailed in specific laboratory logbooks. For a head-to-head comparison of Xpert and RT-MTB assays, lab records were compared. The outcome indicated that the *m*2000 System required various interventions and repeated runs, as well as routine preventive maintenance. A list of technical interventions has been drawn up in Supplementary material (S3).

## Discussion

This study aimed to evaluate the performance of the RT-MTB in a high TB and HIV burden region. To our knowledge, this is the largest study evaluating the RT-MTB diagnostic assay in clinical samples from a unique cohort of HIV-positive patients. We utilized the *m*2000 System for both HIV-1 viral load quantification and MTBC detection, with the purpose of adding evidence for the implementation of high-throughput and multi-disease testing on a single device.

Data on the clinical performance of the RT-MTB for the diagnosis of TB is limited and reported diagnostic values differ across studies (S1). In this clinical study, we compared both, RT-MTB and Xpert assays, to identify MTBC among PLHIV using two different reference standards. For the per-protocol cohort evaluation, the overall RT-MTB sensitivity on decontaminated samples was higher than Xpert. Our findings are in concordance with the study performed by *Scott *et al*. 2017*^22^. Although they obtained higher sensitivity values for Xpert when testing concentrated samples, RT-MTB identified a substantially higher number of MTBC cases among smear negative patients (74.4% *versus* 25.7%). Similar trends were seen in our study, although there was limited statistical power to detect differences when using the composite reference standard (48% *versus* 29% *p* value = 0.05). Analysis of the intention-to-treat cohort did not improve on test parameters, however, RT-MTB showed better sensitivity than Xpert. These results are in line with several studies showing that molecular case-detection diminishes among smear negative patients^5,33^.

On the other hand, Xpert achieved markedly increased specificity and higher PPVs in all analyses. RT-MTB specificity and PPVs values remained lower and constant throughout. Additionally, using the CRS, the specificity of Xpert reached 100% among smear negatives although this could be biased (incorporation bias) by the fact that clinicians were not blinded to Xpert results. In order to get a better understanding of the lower specificity of RT-MTB and if it would be translated into false-positive test results, we evaluated the 22 discordant results among RT-MTB and culture. Six of these cultures were positive for NTM. Cross-reactivity has not been reported previously in similar studies^19,24^, although NTM cultures are often excluded from the analyses or considered as contamination, biasing the study findings^17^. Conversely, in vitro evaluation of RT-MTB showed 97% specificity due to cross-reactivity with two samples containing NTM, although Cn values were close to the established cut-off (Cn = 40)^14^. Importantly, our setting is associated with high NTM isolation in pediatric patients investigated for TB^34^. Our results could therefore indicate some degree of cross-reactivity with NTM. Of the remaining 16 discordant samples, 2 were collected from previous TB patients, 2 were from patients that had been treated recently, 4 were from patients that died after the follow-up period and no other relevant information was found on the remaining 8 discrepant cases. When RT-MTB Cns were compared, the Cn mean among those potential false-positives approached the cutoff of 40 established by the manufacturer. This could support the hypothesis of the detection of either low amounts of DNA in recently treated patients, or real false-positive results. Highly sensitive molecular tests deal with the identification of non-viable DNA^35^ or the detection of cross-contamination during test performance. In a recent meta-analysis to evaluate the laboratory cross-contamination of *Mycobacterium tuberculosis*, 2% of all positive results were found to be false-positives for this reason^36^. Our investigation did not suggest that intra-instrument carry-over was the cause of false positivity. Furthermore, our TB laboratory strictly follows Good Clinical and Laboratory Practice standards (GCLP); thus, cross-contamination of specimens due to material transfer during pre-analytic sample handling appears unlikely.

Since the evaluation of the molecular test performance relies on comparing results to a hypothetical error-free test, using culture as a gold standard brings limitations and possible detection bias^37,38^. Liquid and solid cultures have difficulties in identifying paucibacillary specimens, common in children and HIV patients. Whether positive molecular tests from culture negative samples are false-positives or misclassified real negatives are difficult to disentangle^22^. Therefore, we combined treatment initiation and positive cultures to better evaluate the accuracy of the molecular tests used in this study. The use of composite reference standards has been extensively used not only for tuberculosis^39–41^, but also for other infectious diseases when diagnosis accuracy evaluation might be compromised by weak reference standards^38^. Results on this approach led to improved specificity but lower sensitivity. Microbiological verification of TB cases is still challenging in the diagnostic workup and last year only 56% of cases were bacteriologically confirmed, therefore almost half of tuberculosis patients started treatment based on clinical observation^1^.

Lastly, on the importance of strengthening laboratory diagnosis in all dimension, the theoretical advantages of centralized platforms with greater capacity for testing might be translated into real improvements under operational conditions^42^. Although the Xpert´s PCR is performed in less than 2 hours^12^, just one cartridge can be tested per module, whereas up to 96 tests can be run simultaneously in the *m*2000*sp* platform. Nevertheless, these high-capacity devices, often result in longer turnaround time due to sample preparation and testing, thereby making them inferior in terms of throughput. In addition, they need suitable infrastructure, qualified personnel for the instrument´s set up and the capacity to perform adequate maintenance and handle any technical issues that may arise. The operational challenges (S3) we experienced with the *m*2000*sp* platform, raise awareness on the importance of strengthening diagnosis capacity, not only with regard to more accurate techniques but also on appropriate laboratory infrastructure, resources and trained personnel^7^.

Our study had further limitations. Firstly, we only tested the best quality specimen for liquid and solid culture because of budget concerns. Secondly, we could not assess the diagnostic accuracy of drug susceptibility testing due to a lack of phenotypic resistance strains. One of the advantages of the Abbott assay is its ability to identify rifampicin and isoniazid resistance mutations in the same DNA sample prepared to identify the presence of MTBC. However, we gained information on drug resistance in just 18 specimens with RT-MTB RIF/INH Resistance assay. Twenty-one samples were below the LoD, which is likely due to the lower LoD of the assay (17 CFU/mL) compared to that of the Resistance assay (60 CFU/mL). For the remaining samples, the system reported other test errors. Additionally, a number of technical issues were encountered related to the *m*2000*sp* instrument, leading to repeats and delaying the study. Lastly, the percentage of lost-to-follow-up was higher than expected and we could not characterize false-positive samples any further through other highly sensitive molecular assays or techniques, such as sequencing, in order to assess the true specificity of RT-MTB assay.

## Conclusion

In this study, conducted among PLHIV in southern Mozambique, our results suggest better sensitivity and confirm lower specificity for the Abbott RT-MTB assay compared to the Xpert MTB/RIF. The RT-MTB assay may detect cases that may not otherwise be detected by culture, although this added yield might also be associated with some degree of cross-reactivity with NTMs, detection of non-viable mycobacteria (previously treated patients) or cross-contamination.

The considerable number of “false-positive” results calls for a profound case evaluation on an individual basis, involving trained personnel for the interpretation of molecular results and careful specimen handling to minimize the risk of potential cross-contaminations.

## Supplementary Information

Supplementary Information.

## Data Availability

The datasets generated during the current study are kept at the data center of CISM. An anonymized version of the dataset can be made available upon request to CISM’s Internal Scientific Committee (Email: cci@manhica.net).
