# Supplementary material for: Diagnostic performance of the Abbott RealTime MTB assay for tuberculosis diagnosis in people living with HIV
Source: Sci Rep. 2021 Sep 29;11:19271. doi: 10.1038/s41598-021-96922-3 (PMC8481474; doi:10.1038/s41598-021-96922-3)
Supplement: Supplementary file 1 — Supplementary Information. [file 41598_2021_96922_MOESM1_ESM.pdf]

Diagnostic performance of the Abbott *RealTime* MTB assay for tuberculosis diagnosis in people living with HIV.

Belén Saavedra *[1,2,3], Edson Mambuque[2], Neide Gomes[2], Dinis Nguenha[2], Rita Mabunda[2], Luis Faife[4], Ruben Langa[4], Shilzia Munguambe[2] , Filomena Manjate[2], Anelsio Cossa[2], Lesley Scott[5], Alberto L. García-Basteiro[2,3]

[1] PhD Program in Medicine and Translational Research. Universitat de Barcelona, Barcelona, Spain.

[2] Centro de Investigação em Saude de Manhiça (CISM), Maputo, Mozambique.

[3] ISGlobal, Hospital Clínic - Universitat de Barcelona, Barcelona, Spain

[4] Manhiça Health Research Hospital, Ministry of Health, National Tuberculosis Control Program, Maputo, Mozambique.

[5.] Department of Molecular Medicine and Haematology, School of Pathology, and
iLEAD, Wits Health Consortium
Faculty of Health Sciences, University of the Witwatersrand, Johannesburg, South Africa

* Corresponding author: Belén Saavedra Cervera, belen.saavedra@manhica.net

Supplementary material:

- **S1.Supplementary table 1**
- **S2. Supplementary table 2**
- **S3. Supplementary table 3**

S1.Supplementary table 1. Comparison of diagnostic test parameters within different published studies on the diagnostic accuracy of RT-MTB

Study	Se[1] (%) (95% CI[2])	Sp[3] (%) (95% CI)	Se HIV+[4] (%) (95%CI)	Sp HIV+ (%) (95%CI)	Se sm –[5] (%) (95% CI)	Sp sm – (%) (95% CI)	Se sm+[6] (%) (95% CI)	Sp sm+ (%) (95% CI)	Population & setting	Number of participants and/or specimens	Type of samples
Berhanu et al. 2018	77.8 (64.4-88)	95.6 (91.4-98.1)	68.8 (50-83.9)	95.5 (89.9-98.5)	25.0 (7.27-52.4)	95.6 (91.4-98.1)		-	Presumptive pTB[7] cases (South Africa)	237 participants	Respiratory
Vinuesa et al. 2018	100.0 (81.6-100.0)	100.0 (99.6-100.0)	-	-	-	-	-	-	Presumptive pTB cases (Valencia, Spain)	1020 participants	Respiratory
Tam et al. 2017	-	-	-	-	92.1 (84.5-96.3)	99.8 (98.4-100.0)	98.8 (92.8-99.9)	100.0 (74.7-100.0)	Patients with suspected lower respiratory infection (Hong Kong)	526 participants (610 samples)	Respiratory
Hinic et al. 2017	-	-	-	-	87.5	100.0	100.0	100.0	Presumptive pTB and EPTB[8] (Basel, Switzerland)	287 samples	Respiratory and non-respiratory
Hofmann-Thiel et al. 2016	92.1 (87.9-95)	99.6 (98.3-99.9)	-	-	76.2 (65.4-84.5)		100.0 (97.2-100.0)	-	Presumptive pTB and EPTB) (Gauting, Germany)	715 samples	Respiratory and non-respiratory
Scott et al. 2017	85.5 (74.2-93.1)	92.4 (86.4-96.3)	77.5 (61.5-89.2)	93.1 (86.4-97.2)	-	-	-	-	Presumptive pTB cases (South Africa)	302 participants	Respiratory
Wang et al. 2016	100.0 (98.7-99.9)	90.0 (81.9-95.3)	-	-	-	-	-	-	Presumptive pTB cases TB (China)	270 participants	Respiratory
Chen et al. 2015	-	-	-	-	100.0 (100.0-100.0)	99.3 (97.9-100.0)	100.0 (100.0-100.0)	100.0 (100.0-100.0)	Adult patients with chest symptoms (Hong Kong)	535 samples	Respiratory
Tang et al. 2015	-	-	-	-	81.0		99.0	97.0	specimen bank (FIND)[9] (Geneva, Switzerland)	198 samples	Respiratory

[1]Se: Sensitivity; [2]CI: Confidence Interval ; [3]Sp: Specificity; [4]HIV+: HIV positive patients; [5] sm -: smear negative participants; [6]sm +: smear positive participants; [7]pTB: pulmonary tuberculosis; [8]EPTB: extrapulmonary tuberculosis ; [9]FIND: The Foundation for Innovative New Diagnostics

S2. Supplementary table 2. Details of 22 discrepant results between Abbott RT-MTB and aggregated culture as reference standard

Study code	Xpert MTB/RIF	Culture result	RT-MTB[1]	Cn[2]	Second sample	TB treatment after follow-up period	Death	Old TB[3] Treatment (year)
1001	negative	negative	MTBC[4]	38.77	negative	not	not	yes (1990)
1002	negative	negative	MTBC	38.66	negative	not	not	yes (2006)
1003	negative	negative	MTBC	35.45	not provided	not	not	never
1032	negative	negative	MTBC	37.81	NTM[5]	not	not	never
1042	negative	negative	MTBC	34.68	not provided	not	not	never
2009	negative	negative	MTBC	38.48	not provided	not	not	yes (2016)
2040	negative	negative	MTBC	39.79	negative	not	not	never
2049	negative	negative	MTBC	36.96	negative	not	yes	never
2052	negative	negative	MTBC	38.58	not provided	not	not	never
2056	negative	negative	MTBC	32.62	not provided	not	not	never
2068	negative	negative	MTBC	38.44	negative	not	not	never
2088	negative	negative	MTBC	38.06	not provided	not	not	never
2091	negative	negative	MTBC	39.61	not provided	not	not	never
3047	negative	negative	MTBC	35.57	negative	not	not	never
4009	negative	negative	MTBC	37.68	not provided	not	yes	never
4015	negative	negative	MTBC	37.55	not provided	not	not	never
1009	negative	NTM	MTBC	39.60	not provided	not	not	never
2018	negative	NTM	MTBC	33.96	not provided	not	not	never
2051	negative	NTM	MTBC	36.69	NTM	not	not	never
2090	negative	NTM	MTBC	34.9	not provided	not	not	yes (2016)
3005	negative	NTM	MTBC	32.92	not provided	not	not	never
3006	negative	NTM	MTBC	37.59	not provided	not	yes	never

[1]RT-MTB: Abbott RealTime MTB assay; [2]Cn: mean threshold cycle number ; [3]TB: Tuberculosis; [4]MTBC: Mycobacterium tuberculosis complex;
[5] NTM: non-tuberculous mycobacteria

S3. Supplementary table 3. List of technical interventions

**Technical errors**	*m2000sp*[1] error during sample extraction (code 3310)
	m2000sp error during sample extraction (code 3331)
	m2000sp system block during master mix addition
	m2000rt[2] error in amplification (error for the internal control IC) error internal control (Code 4499)
	m2000sp error during reflex assay(code 4952)
	m2000sp blockage of channel 2
	software failure
**Number of technical interventions required**	9
**Number of run repeated for TB testing**	8
**Number of runs repeated for HIV testing**	4

[1] *m2000sp*: instrument part of the Abbott m2000 RealTime System ; [2] *m2000rt* Real Time PCR system
